# Single Microfluidic Electrochemical Sensor System for Simultaneous Multi-Pulmonary Hypertension Biomarker Analyses

**DOI:** 10.1038/s41598-017-06144-9

**Published:** 2017-08-08

**Authors:** GeonHui Lee, JuKyung Lee, JeongHoon Kim, Hak Soo Choi, Jonghan Kim, SangHoon Lee, HeaYeon Lee

**Affiliations:** 1 KU-KIST Graduate School of Converging Science and Technology, Korea University, Seoul, Republic of Korea; 2 Department of Mechanical and Industrial Engineering, College of Engineering, Northeastern University, Boston, MA 02115 USA; 3 Gordon Center for Medical Imaging, Department of Radiology, Massachusetts General Hospital and Harvard Medical School, Boston, MA 02114 USA; 4 Department of Pharmaceutical Sciences, Northeastern University, Boston, MA 02115 USA; 5 Department of Nano-Integrated Cogno-Mechatronics, Engineering, Pusan National University, Busan, South Korea

## Abstract

Miniaturized microfluidic biosensors have recently been advanced for portable point-of-care diagnostics by integrating lab-on-a-chip technology and electrochemical analysis. However, the design of a small, integrated, and reliable biosensor for multiple and simultaneous electrochemical analyses in a single device remains a challenge. Here, we present a simultaneous microfluidic electrochemical biosensing system to detect multiple biomarkers of pulmonary hypertension diseases in a single device. The miniaturized biosensor, which is composed of five chambers, is precisely and individually controlled using in-house-built pneumatic microvalves to manipulate the flow pathway. Each chamber is connected to an electrochemical sensor designed to detect four different biomarkers plus a reference control. Our design allows for loading of multiple reagents for simultaneous analyses. On the basis of the developed microfluidic electrochemical sensor system, we successfully detected four well-defined pulmonary hypertension-associated biomarkers, namely, fibrinogen, adiponectin, low-density lipoprotein, and 8-isoprostane. This novel approach offers a new platform for a rapid, miniaturized, and sensitive diagnostic sensor in a single device for various human diseases.

## Introduction

Micro total analysis system (µTAS), microfluidic lab-on-a-chip (LOC), and bio-microelectromechanical systems appeared more than 20 years ago to determine, quantify, and separate biomolecules^1^. A microfluidic device is a combination of microchannels and microchambers represented by diverse materials such as polymers, silicon, plastics, and metals^2^. µTAS or LOC has shown great potential in the integration of diverse functional compartments. The miniaturization of microfluidic devices integrated with chemicals and sensors offers a robust method of measurement and detection of ligands/molecules^3^. Obviously, microfluidic integrated devices have numerous advantages, including rapid manipulation of the sample fluid, reduced reagent consumption, cost effectiveness, less labor, and improved sensitivity, along with a wide range of potential applications. Consequently, an explosive growth in integrated microfluidic device has occurred in the last 10–20 years^4, 5^.

Meanwhile, electrochemical sensing plays a key role in detecting biomolecules in a microfluidic device^6, 7^. This sensor uses heterogeneous electron transfer kinetics and is mainly composed of three electrodes: a working electrode (WE) for an analyte contact where an electrochemical reaction takes place, a reference electrode (RE), which allows measurement of the potential of the WE without passing a current through it, and a counter electrode (CE), which allows the current to pass. CE is used to make a connection to the electrolyte to apply current to the WE^8, 9^. Application of electrochemical sensing, which can be achieved through microfabrication technology and microfluidic systems, has gained considerable interest because it is an outstanding candidate for miniaturization^5^. The method of integration of electrochemical sensing and microfluidic systems is simple but exhibits high performance. In addition, this process is ideal for point-of-care diagnostics owing to its high sensitivity, simplicity, and rapid response^5, 10^. Such three-electrode systems can be fabricated in microsize and can cost effectively be combined with a substrate using photolithography or lift-off process^11–13^. In addition, a microfluidic system can be integrated into a microsized substrate to control the environment for multiple electrochemical analyses. Enhancement of efficacy and detection limit is commonly used to demonstrate the performance of an integrated system Thin-film electrode arrays^14^, Au quasi-RE sensors, and other interdigitated array microelectrodes^15^ have been used to improve the detection limit of enzyme-linked electrochemical assay with ease in manipulation, rapid detection, and small-sensor properties. In addition, several groups have developed microfluidic electrochemical LOC using microvalves^16, 17^. For example, microfluidic devices composed of pH indicator and ion-selective electrodes were integrated into electromagnetic valves to control a fluidic device while acting as a micropump^16^. Recently, a microfluidic-valved manipulation system has been developed to analyze DNA hybridization events^17^ in which the accuracy of the DNA hybridization was improved using an automated and controlled system.

Despite their considerable advantages, microfluidic integrated electrochemical biosensors still suffer from limitations such as the use of a limited number of biomarkers in one biosensor for diagnosis, which contains a disposable electrode, and the laborious and time-consuming fabrication process. In addition, confining the fluid to an individual chamber using an integrated microfluidic electrochemical sensor is greatly needed to obtain reliable electrochemical data. Only the use of multiple biomarkers and simultaneous detection of individual biomarkers could resolve these limitations.

In the present study, we developed an electrochemical assay integrated to a microfluidic system to measure multiple biomarkers associated with pulmonary hypertension. By loading a series of biomarkers [fibrinogen, adiponectin, low-density lipoprotein (LDL), and 8-isoprostane] for pulmonary hypertension in a single microfluidic sensor, this system provides a time-effective high-throughput analysis. To our knowledge, our study is the first to evaluate simultaneous multi-detection of pulmonary biomarkers by individually controlling the flow paths in a single device. The proposed LOC system can be applied to practical diagnosis of pulmonary hypertension diseases^18–22^ as an alternative to the conventional enzyme-linked immunosorbent assay.

## Results and Discussion

### Microfluidic electrochemical biosensor fabrication

**Figure 1:**
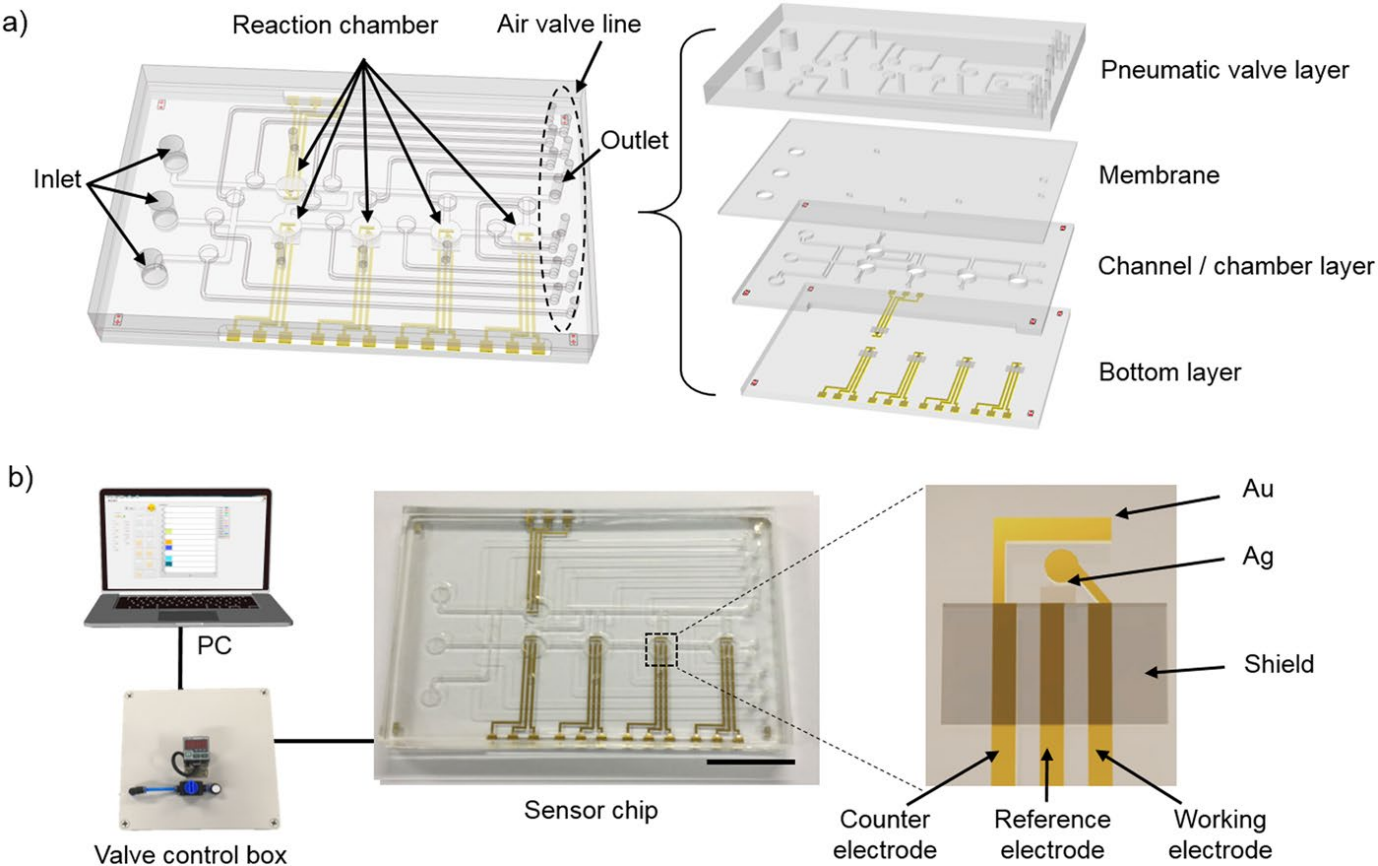
Schematic illustration of the microfluidic system for electrochemical analysis in a single device. (**a**) Schematic description of the microfluidic system integrated electrochemical sensor. The microfluidic electrochemical biosensor is composed of a glass bottom layer with electrodes, PDMS channel/chamber layer, PDMS membrane, and PDMS pneumatic valve layer. (**b**) Schematic diagram of the experimental setup of the microfluidic electrochemical sensor system. The microfluidic electrochemical biosensor is connected to a PC and a control box to enhance transportability (scale bar is 2 cm).

**Figure 2:**
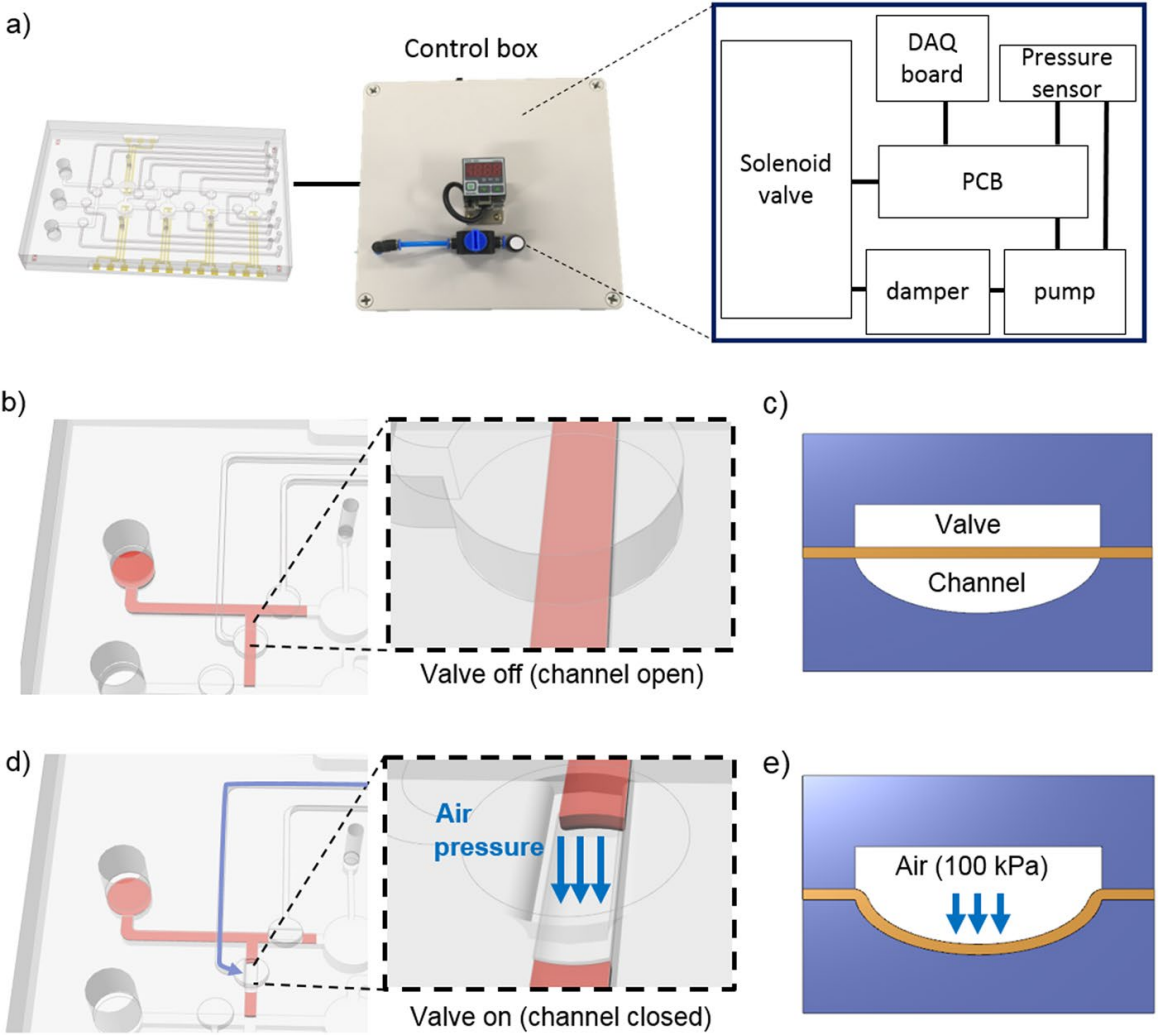
Principle of the on/off pneumatic microvalve. Schematic description of the (**a**) microfluidic electrochemical biosensor with a control box for the pneumatic microvalve, (**b**) valve-on status, (**c**) side view, (**d**) valve-off status, and (**e**) side view.

For successful fabrication of the microfluidic electrochemical biosensor, the microelectrodes and microfluidic polydimethylsiloxane (PDMS) layers were integrated [Fig. 1(a)]. The three-electrode system was adopted for the electrochemical assay, and the microelectrodes were fabricated using stencil lithography (using shadow mask) and electron beam (e-beam) evaporation. One of the common electrode-fabrication processes is the lift-off process^23^. In summary, a sacrificial layer of inverse pattern is deposited on the surface of the substrate. Next, the target material (metal layer) is deposited on the substrate. Then, the remaining sacrificial material is removed. This lift-off process is laborious and time-consuming compared with the stencil lithography. In contrast, we deposited the electrodes at a height of 200 nm using the shadow mask in an e-beam. Therefore, the five electrodes on the glass substrates were easily and rapidly fabricated. A hemicylindrical PDMS microfluidic channel was fabricated to manipulate the fluidic flow pathway (500-µm wide and 200-µm high). The pneumatic microvalves were completely operated under 30 kPa of pneumatic pressure (the digital number of the pressure is displayed on the pressure sensor). We constructed a control box to manipulate the pneumatic microvalves. The microfluidic electrochemical biosensor was connected to the control box, which was connected to a laptop computer [Fig. 1(b)]. The microfluidic electrochemical biosensor chip experiment can be operated without using other additional equipment such as an air-pressure pump, vacuum pump, and microfluidic valve except the electro-power source. The control box contained a micro DC air pump for miniaturization and portability, a pressure sensor to verify the non-leakage status and to control the pump, and 12 solenoid valves for flow path control [Fig. 2(a)]. The pneumatic microvalves were designed to be individually controlled by Labview software [Fig. 2(b–e)].

### Microfluidic channel evaluation

For successful immunoassay using the microfluidic integrated electrochemical biosensor chip, the performance of the integrated system was evaluated using distilled water with inks. The main advantage of our microfluidic electrochemical biosensor is its capability of loading multiple reagents at once. To perform multiple analyses, different types of reagents such as antibodies can be loaded in one device, and the reagents should pass through their individually assigned microchambers. The microfluidic technology integrated electrochemical biosensor chip enables multiple analyses using one chip.

**Figure 3:**
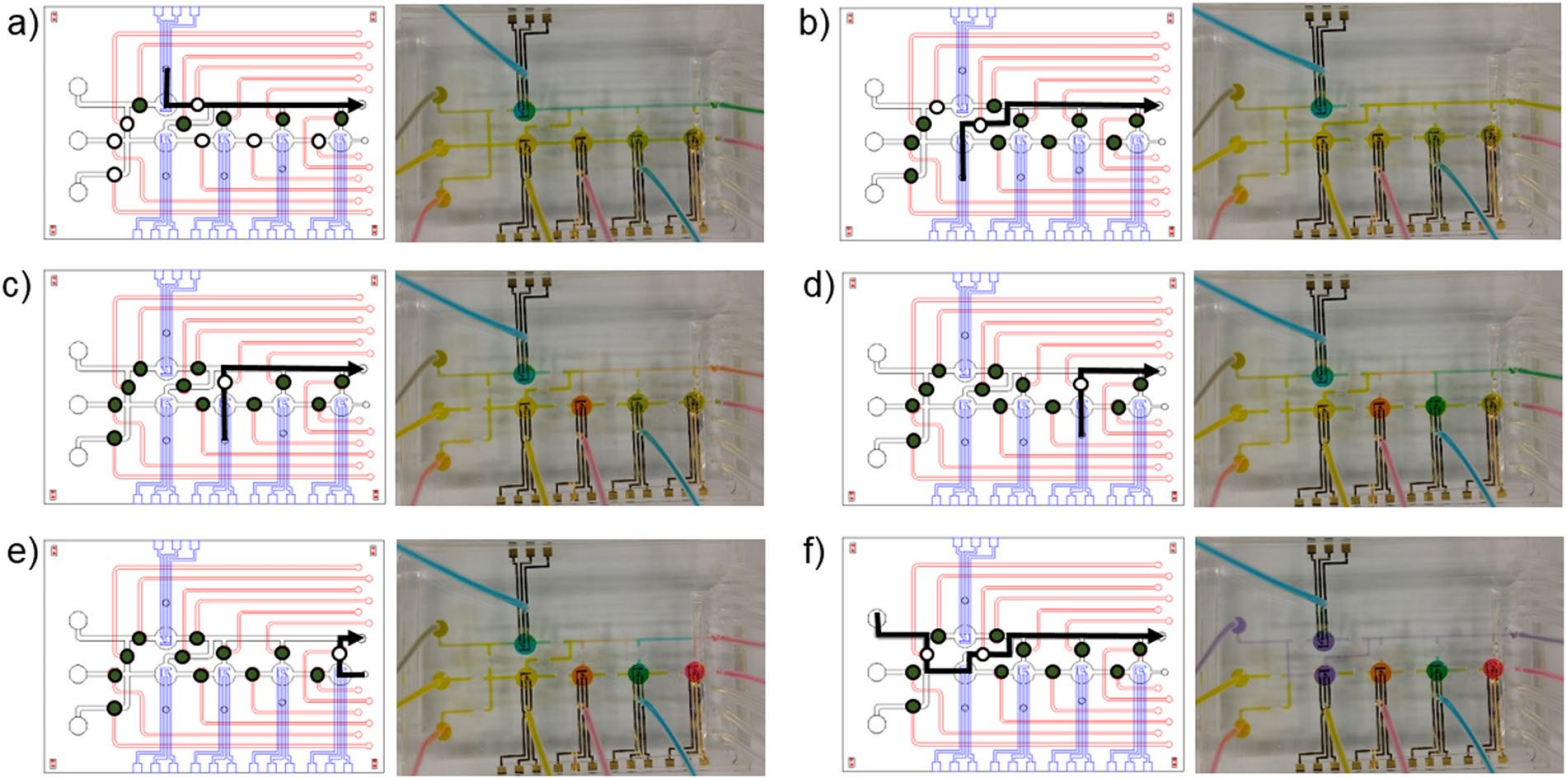
Schematic illustration and photographs of the individual flow control. (**a**–**e**) The arrows indicate the flow pathway in (left) the microfluidic electrochemical biosensor and (right) the distilled water with ink showing the valve performance. Five samples were loaded and filled in each microchamber. This process illustrates the preparation of the immunoaffinity layers. (**f**) Main fluid flow passing through each chamber. This process illustrates that the target material could react in each microchamber.

Five different reagents were loaded in each microchamber [Fig. 3(a–e)], which is connected to their own inlet channels. When a reagent was loaded in a microchamber, it can be drained out of the outlet using pneumatic microvalves without passing through the other microchambers. Then, the target fluid flows into the individual or the entire microchamber [Fig. 3(f)].

To quantify the performance of the pneumatic microvalves, we injected a colored fluid to the closed pneumatic microvalves, and the injected force was measured by a digital force gauge. As a result, the pneumatic microvalves operated until the fluid pressure reached 4.78 N/cm^2^ (Supplementary Fig. 1).

In this microfluidic electrochemical biosensor, multiple inlets can be used for additional purpose. Three different samples can be simultaneously loaded and tested in a single microfluidic electrochemical biosensor. Most of the microfluidic electrochemical biosensors are a single-use and disposable system with a single inlet. Thus, more consumption is required by the microfluidic electrochemical biosensor than our microfluidic system, and it can only analyzed one sample fluid. However, our system with three inlets enables multisample analyses.

### Electrochemical characterization in microfluidic electrochemical sensor system

**Figure 4:**
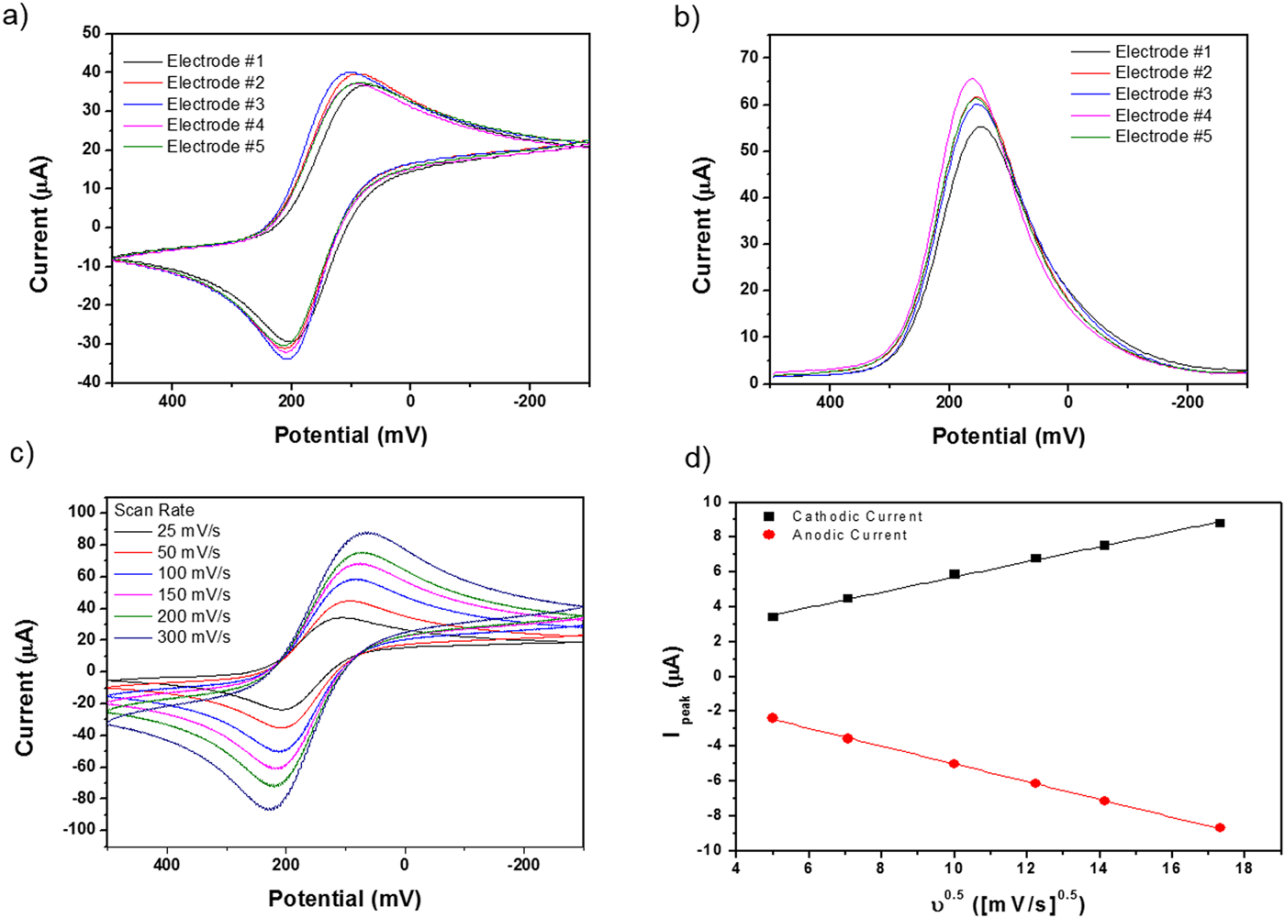
Electrochemical characterization of the microfluidic sensor system. (**a**) CV scans for each of the five electrodes. The scan rate is 50 mV/s. (**b**) SWV scans for each of the five electrodes. (**c**) CV scans of an electrode at 25, 50, 100, 150, 200, 300 mV/s scan rates. (**d**) Influence of the square root of the scan rate on the (black) cathodic and (red) anodic peak currents. The electrolyte is 5 mM K_3_Fe(CN)_6_ + 0.1 M KCl in 10 mM PBS.

The microfluidic electrochemical sensor system in this study consisted of five different electrodes to measure different biomarkers. The electrochemical activity of this system was characterized by cyclic voltammetry (CV) and square-wave voltammetry (SWV) after filling a mediator [(K_3_Fe(CN)_6_)] on each channel. Figure 4(a) and (b) show the CV and SWV results, respectively, of all five electrodes in the system. The mean value and standard deviation among the peak currents of the five different electrodes (*n* = 5) were calculated. The cathodic peak current was 38.33 ± 2.18 µA at 102 mV, and the anodic current was −31.74 ± 2.48 µA at 204 mV in the CV measurements. The peak current in the SWV was 59.95 ± 3.28 µA at 150 mV. The variation in the redox potential in each different electrode was very small, showing only 6.04% degradation. We needed to use electrodes with similar electrochemical properties to obtain accurate signals, and our system can be suitably used as a sensor because each electrode has similar current and potential, as shown by the SWV. We also measured the CV and observed that the cathodic and anodic currents increased with increasing scan rates, as shown in Fig. 4(c). Moreover, the peak currents were proportional to the square root of the scan rate, which is shown in Fig. 4(d). The peak currents yielded linear relationships with 0.43 × 10^−6^ A(s/V)^2^ slope values in the cathodic current and −0.49 × 10^−6^ A(s/V)^2^ slope values in the anodic current.

The peak shape in the CV shows an electrochemical reaction governed by the diffusion of the electroactive species at the surface of the planar electrode^24–26^. Normally, in reversible systems, the peak potential (*E*_pc_ and *E*_pa_) should be consistent with the increasing scan rates because no side reactions take place other than the mass transport of electrons^27–29^. Our electrode shows quasi-reversible reactions because the current flow causes the ohmic resistance to drop near the electrode^30, 31^. After the CV measurement of the fabricated electrode, four different biomarkers of pulmonary hypertension were treated on the electrode to evaluate the biosensing performance of our microfluidic electrochemical sensor system. We mixed four different biomarkers by varying the concentration in the phosphate-buffered saline (PBS). Specific information of the mixture is listed in Supplementary Table 1.

**Figure 5:**
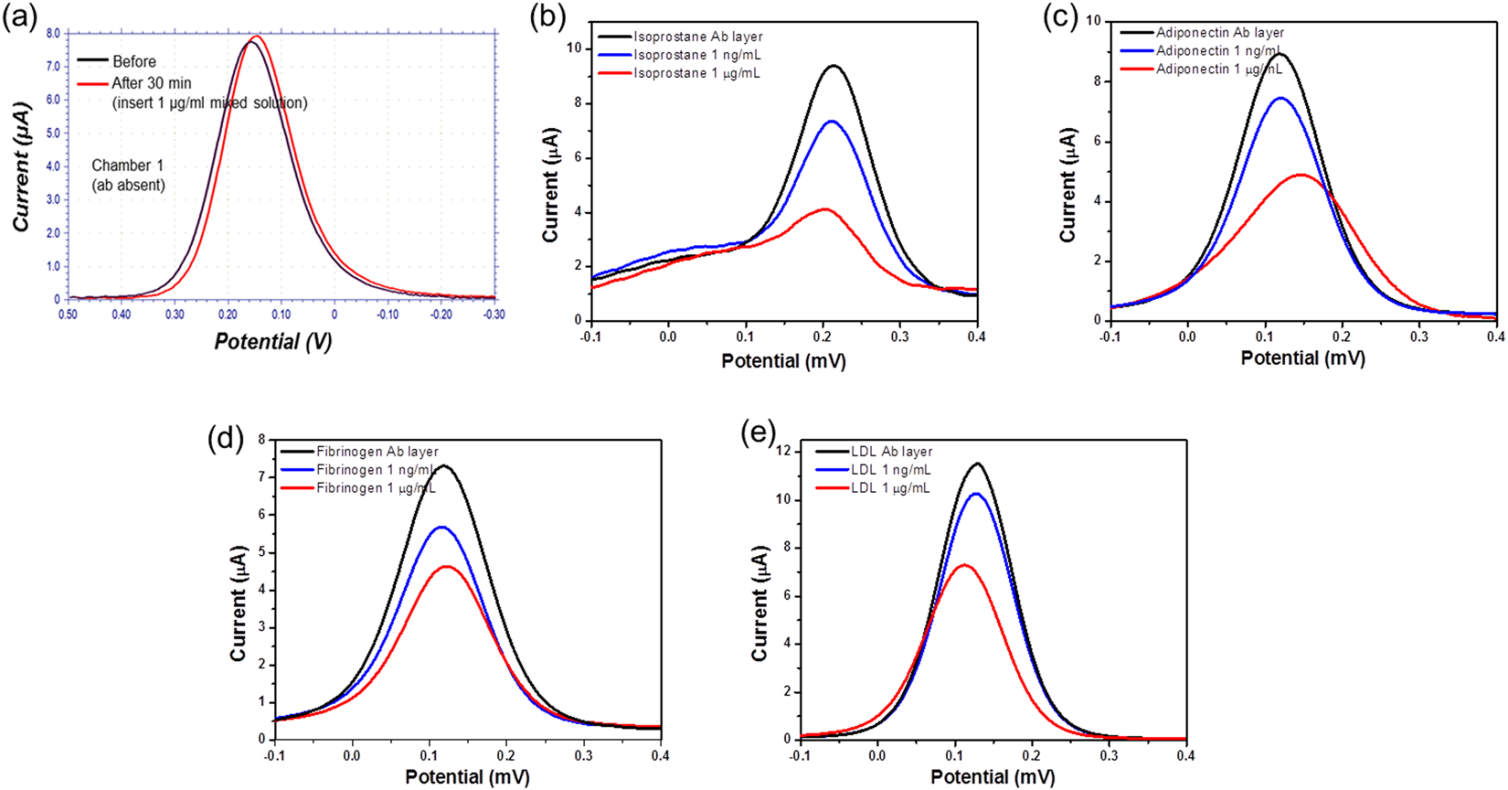
SWV measurements of the current (black solid line) before and after binding of the specific biomarker at (blue solid line) 1 ng/mL and (red solid line) 1 µg/mL concentration. The signal represents the case of (**a**) Chamber 1: reference signal, (**b**) Chamber 2: 8-isoprostane, (**c**) Chamber 3: adiponectin, (**d**) Chamber 4: fibrinogen, and (**e**) Chamber 5: LDL. The electrolyte is 5 mM K_3_Fe(CN)_6_ + 0.1 M KCl in 10 mM PBS.

The CV and SWV signals were measured before and after adding the analyte. Figure 5 shows the electrical signal change after binding each analyte. Chamber 1 [Fig. 5(a)], which was the reference (as negative control) signal, was measured before and after adding Mixture 1 (1 µg/mL concentration of isoprostane + adiponectin + fibrinogen + LDL). Antibody was not treated in this region, and the other site was blocked by bovine serum albumin. Therefore, no antigen could be bound to the electrode surface, which led to the absence of a significant change in Chamber 1. However, from Chambers 2 to 5, specific ab flowed, and the electrodes possessed an immuno-affinity layer to bind each analyte. When we allowed Mixture 2 to flow (1 ng/mL concentration mixed), the SWV peak signal was reduced from 9.3 to 7.2 µA in isoprostane (Chamber 2), from 9.1 to 7.5 µA in adiponectin (Chamber 3), from 7.2 to 5.8 µA in fibrinogen (Chamber 4), and from 3.5 to 3.09 µA in LDL (Chamber 5). In addition, these electrodes are concentration dependent, and we proved this result by loading Mixture 1 (1 µg/ml concentration mixed). The SWV peak signal was reduced from 9.3 to 4.11 µA in isoprostane, from 9.11 to 4.72 µA in adiponectin, from 7.23 to 4.35 µA in fibrinogen, and from 3.51 to 2.27 µA in LDL. When the analyte was bound to the immuno-affinity layer in each of the electrode, it increased the electron-transfer resistance between the electrode and electrolyte [K_3_Fe(CN)_6_] in the solution (Supplementary Fig. 2). These results indicate that the higher concentration of analyte creates a denser and thicker electrical isolation layer, which explains why the redox current decreased after binding to the analyte. In summary, our results demonstrate that the four different biomarkers can be simultaneously detected using only one microfluidic sensor system.

As the employed antibodies demonstrated specificity for isoprostane, adiponectin, fibrinogen, and LDL against other proteins, the tested compounds were mouse Leptin protein to demonstrate selectivity. Supplementary Fig. 3 shows the comparison of the SWV peak responses obtained when different mixtures were loaded, as listed in Supplementary Table 1. No significant effects on the response was shown in the presence of any non-specific binding, thus demonstrating the excellent selectivity of the developed microfluidic sensor for simultaneous isoprostane, adiponectin, fibrinogen, and LDL determination.

## Conclusions

We have developed an integrated microfluidic electrochemical biosensor on a single device for simultaneous multiple analyses in combination with pneumatic microvalves and microfluidic channels. This sophisticatedly designed system provides an easy control of fluidic pathways and individual reagent-loading capability. Pulmonary hypertension biomarkers (fibrinogen, adiponectin, LDL, and 8-isoprostane) were immobilized on separate electrodes, and they were successfully measured at 1 µg/mL concentration. Each chamber was individually controlled during the measurement. The presence of miniaturized flow controller and electrochemical analyzer enhanced the portability and practicality of our proposed sensor for multiple analyses. It showed improved performance of the biosensor because many different biomarkers could be simultaneously measured. In the future, this novel LOC, which integrates an electrochemical system with multiple channels and electrodes, can provide a practical platform for clinical diagnosis.

## Methods

### Fabrication process

#### Microfluidic electrochemical biosensor

Microfluidic PDMS channels were fabricated using standard soft lithography. We used SU-8 100 negative photoresist (MicroChem, USA) to fabricate three different masters: (1) top pneumatic microvalve layer, (2) membrane layer, and (3) microfluidic channel/chamber layer. The SU-8 was spin-coated at 1300 rpm for 40 s on a 4 in silicon wafer with a thickness of 200 µm. To operate the pneumatic microvalves, we fabricated a microfluidic channel with a rounded cross section, as previously described^32^. The PDMS channel layer, composed of microscale holes with rectangular cross section, was bonded to a thin PDMS membrane (10 µm thick) using oxygen plasma treatment for 30 s (600 mTorr, 60 W). The membrane was deformed to a hemicylindrical form by applying negative pressure through the hole. Then, SU-8 2025 solution was applied to the deformed PDMS membrane and cross-linked by UV exposure. Next, we fabricated the three PDMS layers using PDMS prepolymer (mixture of 10:1 silicon elastomer and curing agent) on an SU-8 master mold, followed by alignment of the pneumatic microvalve, membrane, and channel/chamber using oxygen plasma treatment for 30 s (600 mTorr, 60 W).

#### Electrodes

The microelectrodes were fabricated using a shadow metal mask (Youngiin Astech, Korea) and an e-beam evaporator (SNTek, Korea). A clean slide glass substrate (76 × 52 mm) was placed in an e-beam chamber with an aligned shadow metal mask. A patterned Au layer (2000 Å) was then deposited following an adhesion layer of Ti (50 Å) according to the manufacturer instructions (Youngiin Astech, Korea)^33^. Next, the second shadow mask was aligned to a pre-patterned substrate. Then, an Ag layer (2000 Å) was deposited using an adhesion layer of Ti (50 Å). To maximize the sensitivity of the microfluidic electrochemical biosensor, a shield layer was deposited using a standard photolithography process. SU-8 5 (MicroChem, USA) was spin-coated on the glass surface at 1000 rpm for 30 s and baked on a hotplate for 3 min at 90 °C. The substrate was finally developed and rinsed using an SU-8 developer (MicroChem, USA) after the UV crosslinking. Each chamber was composed of three parts: CE, RE, and WE.

#### Pneumatic microvalve control system

The pneumatic microvalve control box was designed to control the microfluidic pathway. In summary, the control box was composed of a micro DC air pump (DAP-370P-12V, Motor Bank, Korea), a damper case, a solenoid valve (V290-4E, Shinyeong Mechatronics, Korea), a solenoid bracket (MN290-12-C4, Shinyeong Mechatronics), a pressure sensor (Autonics, Korea), and a data acquisition board (USB-6501, National Instruments, USA). Tygon tubes were connected as solenoid valves to the microfluidic sensor, and the pneumatic microvalves were controlled by Labview software (National Instruments).

### Measurement process

Preparation of the immunoaffinity layer. The fibrinogen, biotinylated anti-fibrinogen antibody, oxidized LDL, biotinylated anti-oxidized LDL antibody, adiponectin, and biotinylated anti-adiponectin antibody were purchased from Abcam (Cambridge, USA). The 8-isoprostane and anti-8-isoprostane antibody were obtained from Oxford Biomedical Research (Rochester Hills, USA). The streptavidin, ethanolamine hydrochloride, 11-mercaptoundecanoic acid (11-MUA), N-(3-dimethylaminopropyl)-N′-ethylcarbodiimide (EDC), N-hydroxysuccinimide (NHS), and potassium ferricyanide [K_3_Fe(CN)_6_] were purchased from Sigma–Aldrich (St. Louis, USA). All chemicals used were of analytical grade and used without further purification.

All microfluidic electrodes in the microfluidic channel were treated with acetone and cleaned using ethanol and deionized water before use. After drying in a stream of N_2_, self-assembled monolayers (SAMs) were prepared on the electrodes by incubating them in 10 mM 11-MUA dissolved in anhydrous ethanol for 1 h at room temperature. Next, 50 mM EDC and 50 mM NHS in a sodium acetate buffer (pH = 5.5) were used to activate the NHS ester functional groups for 15 min. The streptavidin (10 µg/mL in PBS) was then immobilized on the SAMs for 30 min at room temperature. Then, the unreacted functional ester groups were blocked by 1 M ethanolamine for 30 min. Finally, a solution of 10 µg/mL biotinylated antibody was immobilized using the biotin–streptavidin affinity^34^. Each biomarker with a concentration of 1 µg/mL was loaded for 30 min and attached to an electrode using a micropipette via the reaction chamber inlets under the control of pneumatic microvalves to isolate each biomarker in one reaction chamber (Chambers 2–5). Then, the reaction chamber inlets were covered with a piece of PDMS to prevent leakage. The biomarker on the first electrode was left untreated to serve as a “reference” signal to check the overall performance. Prior to the addition of antigen, bovine serum albumin (1 mg/mL) was used to fill the microfluidic chamber to block the non-specific binding of bio-conjugates on all surfaces. Subsequently, 1 µg/mL of the four mixed analytes (fibrinogen, adiponectin, LDL, and 8-isoprostane) was loaded to all chambers for 30 min. Washing using PBS and drying using air gun were applied between every process. All chemicals flowed at a rate of 20 µL/s during the application of the microfluidic electrochemical biosensor.

#### Electrochemical measurement

After the immobilization process, the electrode was thoroughly washed using PBS (pH = 7.4). The washing buffer flowed through the main channel for 5 min to remove non-specific binding proteins. After the washing process, the electrolyte (5 mM K_3_Fe(CN)_6_ + 0.1 M KCl in PBS (pH = 7.4) flowed to each chamber via the main channel for 3 min. All electrodes are connected to the multichannel potentiostat (1040 C, CH instruments, Texas) before the measurement was started. After the electrolyte completely filled each chamber, CV and SWV were performed at room temperature. CV was also performed from 0.5 to −0.3 V at 50 mV/s scan rates. The SWV scan was conducted from −0.3 to 0.5 V with a 25 mV amplitude at a 4 mV increment for simultaneous quantitative measurement of the biomarkers.

## Electronic supplementary material

Supplementary information
