# Supplementary material for: Single Microfluidic Electrochemical Sensor System for Simultaneous Multi-Pulmonary Hypertension Biomarker Analyses
Source: Sci Rep. 2017 Aug 8;7:7545. doi: 10.1038/s41598-017-06144-9 (PMC5548735; doi:10.1038/s41598-017-06144-9)
Supplement: Supplementary file 1 — Supplementary information [file 41598_2017_6144_MOESM1_ESM.pdf]

Single Microfluidic Electrochemical Sensor System for Simultaneous Multi-Pulmonary Hypertension Biomarker Analyses

GeonHui Lee[1†], JuKyung Lee[2†], JeongHoon Kim[1], Hak Soo Choi[3], Jonghan Kim[4], SangHoon Lee[1] and HeaYeon Lee[4,5*]

[1]KU-KIST Graduate School of Converging Science and Technology, Korea University, Seoul, Republic of Korea.

[2]Department of Mechanical and Industrial Engineering, College of Engineering, Northeastern University, Boston, MA 02115, USA.

[3]Gordon Center for Medical Imaging, Department of Radiology, Massachusetts General Hospital and Harvard Medical School, Boston, MA 02114, USA.

[4]Department of Pharmaceutical Sciences, Northeastern University, Boston, MA 02115, USA.

[5]Department of Nano-Integrated Cogno-Mechatronics, Engineering, Pusan National University, Busan, South Korea.

*Correspondence to H.Y.L (he.lee@neu.edu)

†These authors contributed equally to this work.

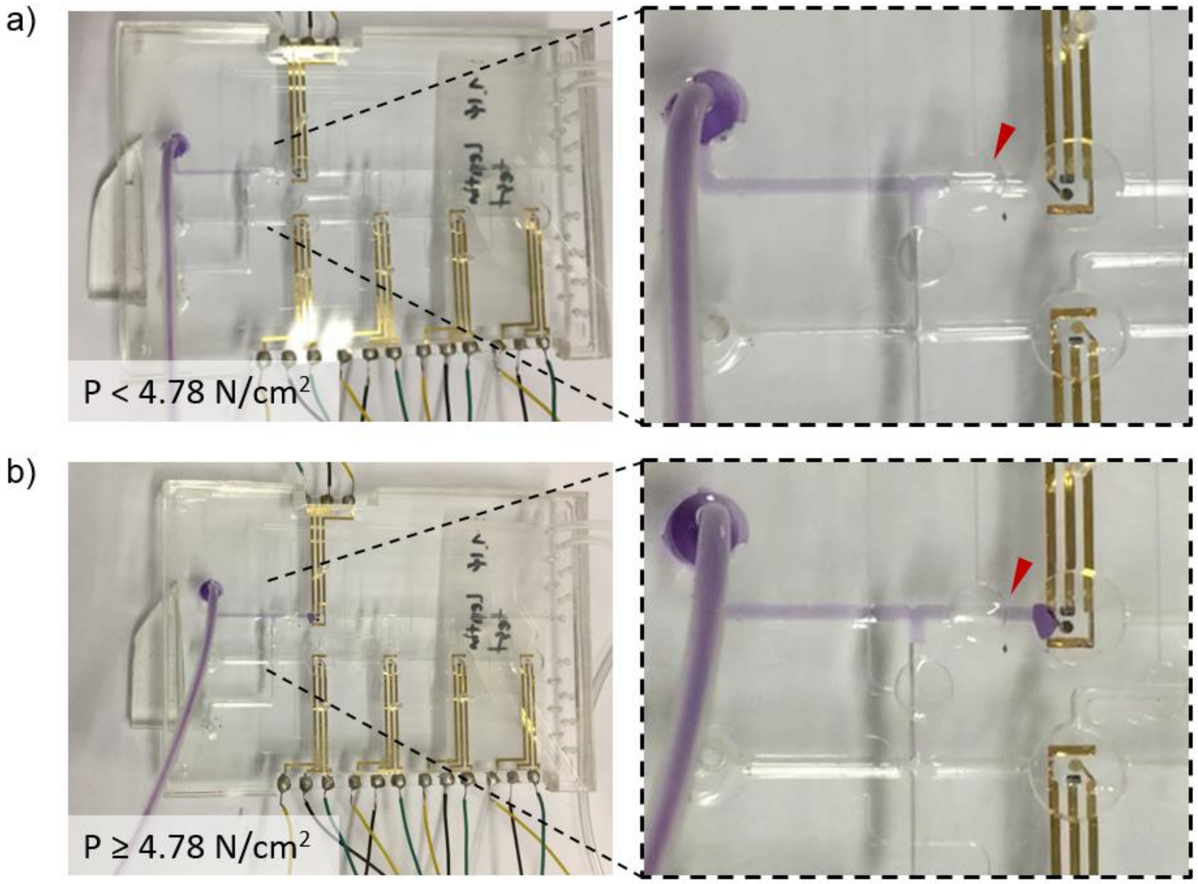

Supplementary Fig. 1. Performance test of the pneumatic valve. To quantify the performance of the pneumatic valves, a colored fluid was injected to the closed pneumatic valves, and the injected force was measured by a digital force gauge. The pneumatic valves operated until the fluid pressure reached 4.78 N/cm^2.

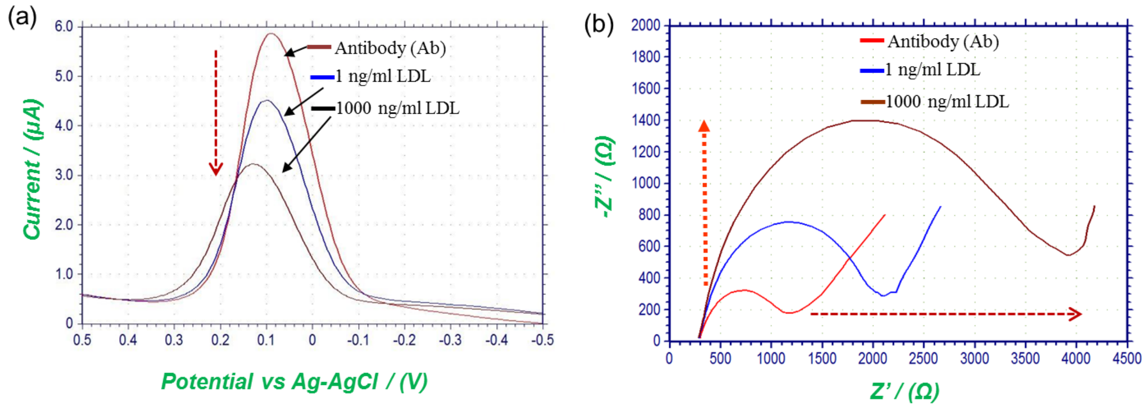

Supplementary Fig. 2. (a) SWV, (b) impedance measurement of biotinylated Ab with 1 ng/mL (low concentration) binding and with 1000 ng/mL (high concentration) binding.

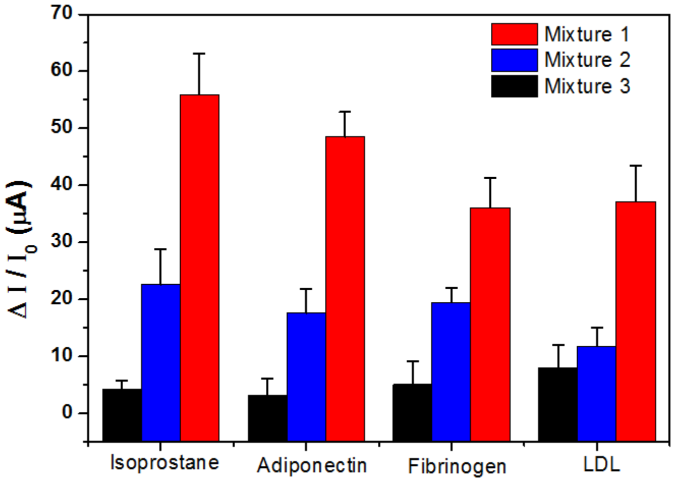

Supplementary Fig. 3. SWV responses of the presence of 1 μg/mL mouse leptin (black bar: non-specific binding protein), 1 ng/mL mixed solution (blue bar), 1 μg/mL mixed solution (red bar). (n=3)

	Mixture of antigen injected into main channel				
	Isoprostane	Adiponectin	Fibrinogen	LDL	Leptin
Mixture 1	1 μg/mL	1 μg/mL	1 μg/mL	1 μg/mL	0
Mixture 2	1 ng/mL	1 ng/mL	1 ng/mL	1 ng/mL	0
Mixture 3	0	0	0	0	1 μg/mL

Supplementary Table. 1. The mixtures of isoprostane, adiponectin, fibrinogen, LDL in the three different injected solutions for evaluating sensitivity and selectivity of each immunosensor fabricated in the microchannel.
